# Platelet adhesion on commercially pure titanium plates in vitro III: effects of calcium phosphate-blasting on titanium plate biocompatibility

**DOI:** 10.1186/s40729-020-00270-2

**Published:** 2020-11-20

**Authors:** Masayuki Nakamura, Hachidai Aizawa, Hideo Kawabata, Atsushi Sato, Taisuke Watanabe, Kazushige Isobe, Yutaka Kitamura, Takaaki Tanaka, Tomoyuki Kawase

**Affiliations:** 1 Tokyo Plastic Dental Society, Kita-ku, Tokyo, Japan; 2 Department of Materials Science and Technology, Niigata University, Niigata, Japan; 3 Division of Oral Bioengineering, Institute of Medicine and Dentistry, Niigata University, Niigata, Japan

**Keywords:** Platelet-rich plasma, CD62P, CD63, Titanium, Implant, Calcium phosphates, Blasting

## Abstract

**Background:**

Platelet-rich plasma (PRP) is often used to improve surface biocompatibility. We previously found that platelets rapidly adhere to plain commercially pure titanium (cp-Ti) plates in the absence, but not in the presence, of plasma proteins. To further expand on these findings, in the present study, we switched titanium plates from a plain surface to a rough surface that is blasted with calcium phosphate (CaP) powder and then examined platelet adhesion and activation.

**Methods:**

Elemental distribution in CaP-blasted cp-Ti plates was analyzed using energy-dispersive X-ray spectroscopy. PRP samples prepared from anticoagulated blood samples of six healthy, non-smoking adult male donors were loaded on CaP-blasted cp-Ti plates for 1 h and fixed for examination of platelet morphology and visualization of PDGF-B and platelet surface markers (CD62P, CD63) using scanning electron microscopy and fluorescence microscopy. Plain SUS316L stainless steel plates used in injection needles were also examined for comparison.

**Results:**

Significant amounts of calcium and phosphate were detected on the CaP-blasted cp-Ti surface. Platelets rapidly adhered to this surface, leading to higher activation. Platelets also adhered to the plain stainless surface; however, the levels of adhesion and activation were much lower than those observed on the CaP-blasted cp-Ti plate.

**Conclusions:**

The CaP-blasted cp-Ti surface efficiently entraps and activates platelets. Biomolecules released from the activated platelets could be retained by the fibrin matrix on the surface to facilitate regeneration of the surrounding tissues. Thus, PRP immersion could not only eliminate surface air bubbles but also improve the biocompatibility of the implant surface.

## Introduction

Platelet-rich plasma (PRP) is now widely applied in various fields of regenerative medicine. Its clinical application is based on the evidence that platelets concentrated in PRP provide high levels of growth factors along with fibrinogen/fibrin, both of which are involved in the tissue engineering triangle and are expected to function cooperatively in successful tissue regeneration [1]. PRP is often used to treat the implant surface prior to implantation. The primary purpose of this process is to physically remove air bubbles, thus improving biocompatibility [2]. Other purposes include functionalization of the implant surface with the biomolecules included in PRP preparations. However, no rigorous evidence has emerged to endorse the latter purpose yet.

In the past two decades, surface modification has been the main target of research and development on implant fabrication. To enlarge the surface free area for bone and osteoblast adhesion and thereby reinforce the initial stability of the implants, the implant surface is usually roughened using chemical, electrochemical, and/or mechanical techniques [3]. Among these techniques, blasting is a relatively simpler and more convenient technique, and thus has been applied widely in the implant manufacturing industry. Ishikawa et al. first reported that blasting with hydroxyapatite (HA) powders for the surface modification of implants efficiently coats HA on the titanium (Ti) implant surface [4]. The blasting technique used in this study adopts calcium phosphate (CaP) powder (HA:β-tricalcium phosphate = 1:4) as an abrasive [5, 6]. The attachment and retention of CaP on the Ti surface improves its biocompatibility and induces osseointegration by virtue of its high osteoconductivity [7–10].

Till date, many studies have investigated osteoblast behavior on Ti surfaces [11–14]. Judging from the low blood compatibility, it is predictable that platelets are activated to attach and aggregate on the Ti surface. In case of non-anticoagulated blood, it is believed that fibrin networks are formed on the Ti surface to induce the adhesion of platelets initially, followed by osteoblasts or mesenchymal stem cells next [3, 15]. This process is thought to significantly influence osseointegration and the subsequent mineralization process. In previous studies [2, 16], we examined platelet behavior on the plain cp-Ti surface in case of anticoagulated blood and found that the adhesion of platelets served in the form of PRP onto the cp-Ti surface is significantly suppressed by plasma proteins, such as albumin. Thus, we concluded that PRP immersion may not be as helpful as expected in the functionalization of the implant surface. However, this conclusion is based on the data obtained from plain cp-Ti surfaces, and it is possible that platelets may act differentially on a rough surface. The rough surface model used in the present study mimics a certain type of implant commercially available in the clinical setting.

To expand our previous findings, in the present study, we examined and compared platelet adhesion and activation on CaP-blasted cp-Ti and plain SUS316L stainless steel plates. The plain SUS316L stainless steel plates were used as a model of injection needles used for blood collection and subsequent PRP preparation. Since both CaP-coating and roughness increase protein adsorption and cell adhesion [17, 18], we initially raised a working hypothesis that the CaP-blasted cp-Ti plate may capture and retain a greater number of platelets, even in the form of liquid PRP, than the plain cp-Ti plate. However, although albumin and other plasma components are present in the form of PRP, the ability of CaP-blasted cp-Ti plates to trap and retain platelets was much higher than expected.

## Materials and methods

### Preparation of pure PRP

Blood samples were collected from six non-smoking, healthy, male volunteers aged 46–62 years. Despite having lifestyle-related diseases and taking medication, these donors, that is, our team members and our relatives, had no limitations on the activities of daily living. These donors were also declared to be free of HIV, HBV, HCV, or syphilis infections. In addition, a prothrombin test was performed on all the blood samples by means of CoaguChek® XS (Roche, Basel, Switzerland), and all the samples were found to be normal. The study design and consent forms for all the procedures (project identification code: 2297) were approved by the Ethics Committee for Human Participants at the Niigata University School of Medicine (Niigata, Japan) and complied with the Helsinki Declaration of 1964, as revised in 2013.

First, ~ 9 mL of peripheral blood was collected in plain glass vacuum blood collection tubes (Vacutainer**®**; Beckton & Dickinson, Franklin Lakes, NJ, USA) containing 1.5 mL of a formulation of acid-citrate-dextrose (ACD-A) [2, 16]. Whole-blood samples were stored in a rotating agitator at ambient temperature and were used within 24 h. Thereafter, the samples were centrifuged at 402×*g* for 8 min (soft spin). The upper plasma fraction, ~ 2 mm beyond the interface between the plasma and red blood cell fractions, was then transferred into 2-mL sample tubes and centrifuged once again at 1065×*g* for 3 min (hard spin) to collect the resting platelet pellets. Afterwards, the platelets were resuspended in acellular plasma to adjust the platelet concentration to 3.0 × 10^5^/μL.

Platelets and other blood cell counts were measured using a pocH 100iV automated hematology analyzer (Sysmex, Kobe, Japan). The exclusion of white and red blood cells was confirmed in pure PRP preparations prior to the following experiments.

### CaP-blasted cp-Ti plates and platelet inoculation

cp-Ti plates (Nilaco, Tokyo, Japan) were blasted with a mixture of HA and β-TCP (in a ratio of 1:4) at 0.3 MPa. The resulting cp-Ti plates were cut into small, square 10 × 10 mm^2^ pieces; washed serially with acetone (60 s), ethanol (2 × 60 s), and distilled water (2 × 60 s) in an ultrasonic cleaner (Citizen, Tokyo, Japan); and air-dried.

Platelet suspensions (3 × 10^7^ per plate) were inoculated onto CaP-blasted plates and the plates were incubated at ambient temperature for 60 min. The CaP-blasted plates were then vigorously washed two times with phosphate-buffered saline (PBS) on a shaker (~ 10 s) and subjected to spectrophotometric assay without fixation or with 10% neutralized formalin fixation for staining. For energy dispersive X-ray spectroscopy (EDS) (JCM-6000 with JED-2300; JEOL, Akishima, Japan) and scanning electron microscopy (SEM) (TM-1000; Hitachi, Tokyo, Japan) examination, the samples were fixed with 2.5% glutaraldehyde and dehydrated serially, as described below.

Since we vigorously examined plain cp-Ti plates in the previous studies [2, 16], we adopted plain SUS-316 stainless steel plates (Nilaco) for comparison with the CaP-blasted cp-Ti plates. This choice was based on the routine preparation of PRP by many clinicians and operators in private practice using syringes equipped with needles usually composed of SUS316L. To evaluate platelet loss and activation in the preparation process, immersion in PRP and examination of adherent platelets were performed, as detailed below.

### Plate examination using SEM

To evaluate their surface roughness, both plate types were examined via SEM and atomic force microscopy (AFM). The surfaces of the plates were coated with gold-palladium using a sputter coater (MSP-1S; Vacuum Device, Mito, Japan) and examined using SEM (Hitachi) operated at an accelerating voltage of 15 kV [11, 19]. To examine platelet adhesion and morphology, the platelets adhering to the plates were fixed with 2.5% neutralized glutaraldehyde, dehydrated, and freeze-dried as described previously [20, 21].

### Plate examination using AFM

To evaluate the surface roughness, both plate types were directly subjected to AFM examination. The AFM height images of the samples were recorded in alternating current (AC) mode at room temperature in PBS using the NanoWizard 3 (JPK Instruments AG, Berlin, Germany) AFM system. Soft cantilevers (contact-G; BudgetSensors, Sofia, Bulgaria) were used for scanning through the quantitative imaging mode [2, 11].

### Elemental examination and mapping using EDS

For elemental analysis and mapping of CaP-blasted Ti plates, platelet samples were prepared, fixed, dehydrated, and sputter-coated with Au-Pd (vacuum device) using SEM with an EDS detector (JEOL) at an acceleration voltage of 10 kV. Cell-free samples were directly sputter-coated with gold-palladium and used for EDS examination. The data provided the elemental composition from the surface to a depth of 1 μm under the conditions recommended by the manufacturer [11].

### Quantitative and qualitative determination of adherent platelet counts

Platelets were inoculated onto the surface of CaP-blasted Ti and plain SUS316 plates and incubated for 60 min. The plates were then washed with PBS to remove non-adherent platelets and were further incubated for 2 h with a highly water-soluble tetrazolium dye (Cell Counting Kit-8; Dojindo, Kumamoto, Japan). After incubation, 100 μL of the supernatant was collected and its absorbance was measured using a microplate reader (Model 680; Bio-Rad, Hercules, CA, USA) at a wavelength of 450 nm (reference: 570 nm) [2].

Alternatively, platelets were fixed with 10% neutralized formalin, microperforated with 0.1% Tween-20-containing PBS (T-PBS) for 1 min, and stained with phalloidin (Cytopainter Phalloidin-iFlour 555 Reagent; Abcam, Cambridge, MA, USA) at ambient temperature in the dark and observed under a fluorescence microscope (ECLIPSE 80i; Nikon, Tokyo, Japan) connected to a cooled CCD camera (VB-7000; Keyence, Osaka, Japan). The occupancy of adherent platelets was determined using image analysis software (WinROOF version 6.0, Mitani Corp., Fukui, Japan). In brief, three images were randomly selected from each sample. After RGB separation and manually optimizing the threshold, the images were binarized [19], and the apparent areas covered by platelets per region of interest were calculated. The data were obtained from a single representative experiment.

### Immunocytochemical fluorescence staining

The CaP-blasted Ti and plain SUS316L plates were washed with PBS and the fixed platelets were microperforated with T-PBS (0.1% Tween-20-containing PBS) for 1 min [16]. The samples were washed twice with PBS and blocked with 0.1% Block Ace (Sumitomo Dainippon Pharma Co. Ltd., Osaka, Japan) in T-PBS for 1 h. The samples were then treated with mouse monoclonal anti-CD62P, anti-CD63 (1:100 dilution; BioLegend, San Diego, CA, USA), or anti-PDGF-B (1:200 dilution; Santa Cruz Biotechnology, Dallas, TX, USA) antibodies overnight at 4 °C. Post-treatment, the samples were again washed twice with T-PBS and subsequently probed with a secondary antibody (goat anti-mouse IgG H&L conjugated with Alexa Fluor**®** 488; Abcam, Cambridge, MA, USA) for 60 min along with phalloidin (Cytopainter Phalloidin-iFlour™ 555 Reagent; Abcam) at ambient temperature in the dark. Isotype controls for mouse primary antibodies (Abcam) were used as negative controls.

Finally, after washing with PBS, the samples were mounted using an antifade mounting medium (Vectashield**®**; Vector Laboratories, Burlingame, CA, USA), and target proteins were examined under a fluorescence microscope (Eclipse 80i; Nikon, Tokyo, Japan) connected to a cooled CCD camera (VB-7000; Keyence, Osaka, Japan) [22].

### Visualization of polyphosphates

Polyphosphates stored in platelets and released from platelets were visualized using 4′,6-diamidino-2-phenylindole (DAPI; Dojindo, Kumamoto, Japan). PRP was loaded on the plates, as described above, washed, and fixed with 10% neutralized formalin for 30 min. The platelets were then treated with 0.01 g/mL DAPI- and phalloidin-containing PBS for 15 min and subjected to microscopic examination using a fluorescence microscope (Eclipse 80i; Nikon) equipped with a BV-2A filter cube (excitation filter: 400–440 nm; dichroic mirror: 455 nm; barrier filter: 470 nm) and a G-2A filter cube (excitation filter: 510–560 nm; dichroic mirror: 575 nm; barrier filter: 590 nm) to detect DAPI and phalloidin, respectively.

### Contact angle analysis

The contact angles of the CaP-blasted cp-Ti and SUS316L platelets were measured using a contact angle meter (LSE-ME1; Nick Corp., Kawaguchi, Japan) to determine the time-course changes in the surface wettability [11]. Each plate was cleaned with acetone, rinsed with ethanol, dried as described above, and subjected to contact angle analysis using distilled water for up to 15 min.

### Statistical analysis

Data are expressed as the mean ± standard deviation. For two-group comparisons, a Mann-Whitney rank sum test was performed to compare mean values (SigmaPlot 13.0; Systat Software, Inc., San Jose, CA, USA). For multigroup comparisons, according to the suggestion based on the results of both normality and equal variance testing, a one-way ANOVA followed by Bonferroni’s multiple-comparisons test was performed to compare the mean values (SigmaPlot 13.0). *P* < 0.05 was considered statistically significant.

G*Power software (version 3.1.9.7. Heinrich-Heine-Universität Düsseldorf, Düsseldorf, Germany) was used to calculate the power of the statistical analyses and confirm the validity of the statistical analyses. For the data shown in Figs. 6 and 8, the power values were 1.00.

## Results

**Fig. 1:**
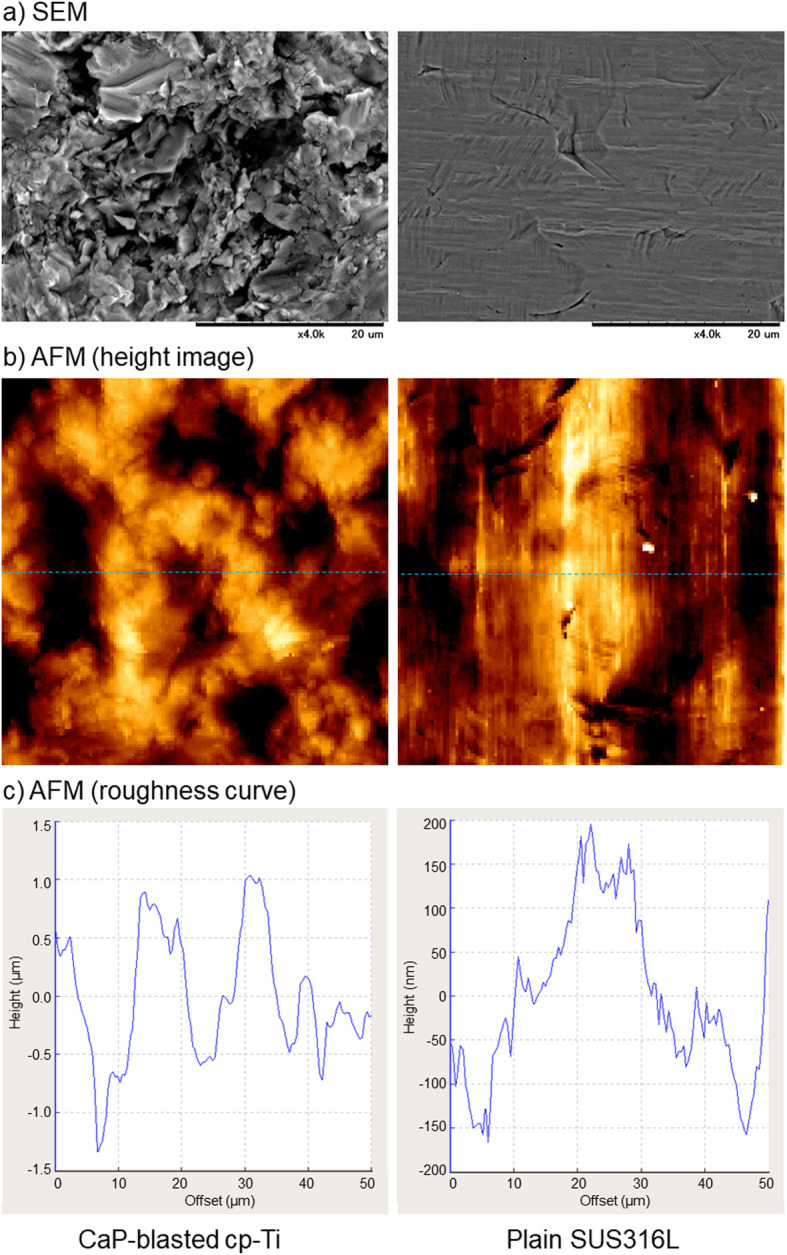
Surface microstructure of CaP-blasted cp-Ti and plain SUS316L plates. **a** The plates were directly sputtered without fixation or dehydration process and subjected to SEM examination. **b** The plates were directly subjected to AFM examination. Size of the height images is 50 × 50 μm. **c** Roughness profiles were drawn from the height images (blue dashed lines)

Figure 1 shows the surface roughness of CaP-blasted cp-Ti and plain SUS316L plates. The surface of the CaP-blasted cp-Ti plate was substantially rough, whereas that of the SUS316L plate was only roughened slightly. The maximum heights (from the bottom of concave to the top of convex) in these images were approximately 2.3 μm (CaP-blasted cp-Ti) and 350 nm (SUS316L).

**Fig. 2:**
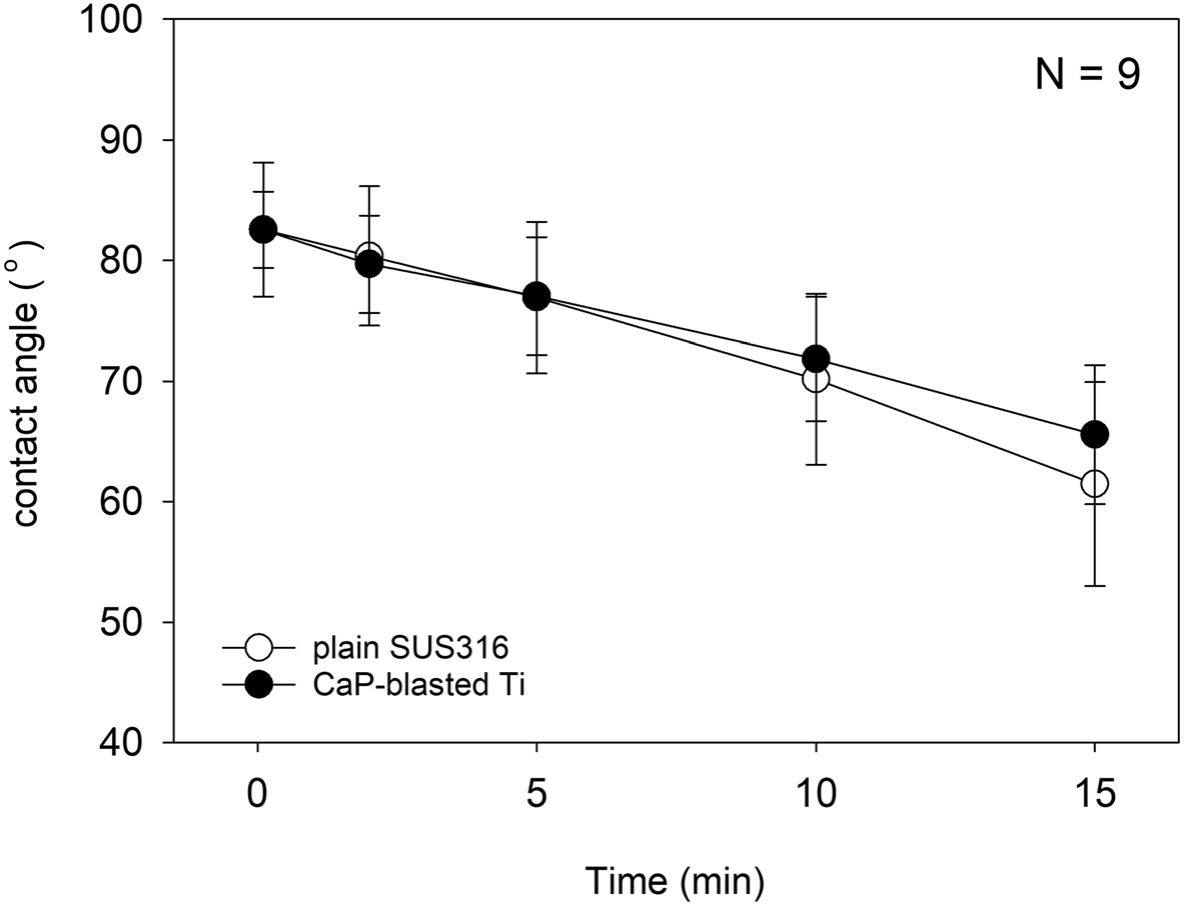
Time-course changes in contact angles of CaP-blasted cp-Ti and SUS316L surfaces. There was no statistical difference between these surfaces at any of the time points. *N* = 9

Figure 2 shows time-course changes in the contact angles of CaP-blasted cp-Ti and SUS316L surfaces. For up to 15 min, the contact angles of both the plates declined from approximately 82 to 63 [CaP-blasted cp-Ti: 82.5 ± 3.2° (10 s), 79.7 ± 4.1° (2 min), 77.1 ± 4.9° (5 min), 71.8 ± 5.2° (10 min), 65.6 ± 5.8° (15 min)] [SUS316L: 82.6 ± 5.6° (10 s), 80.4 ± 5.8° (2 min), 76.9 ± 6.3° (5 min), 70.2 ± 7.1° (10 min), 61.5 ± 8.5° (15 min)]: similarly in a time-dependent manner, and significant differences between these plates were not observed at any of the time points.

**Fig. 3:**
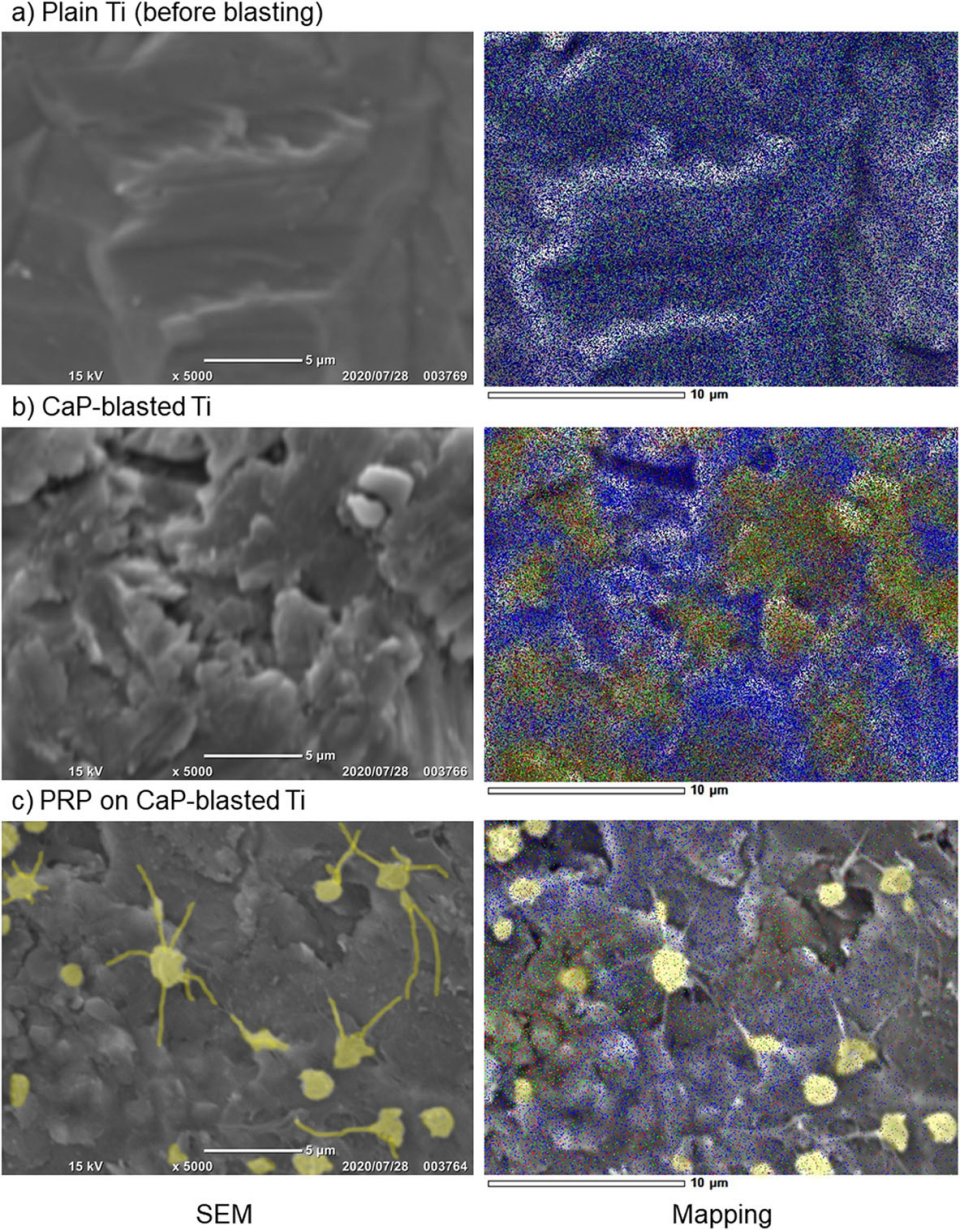
Elemental mapping of plain and CaP-blasted cp-Ti plate surfaces. The **a** plain and **b** CaP-blasted cp-Ti plates were directly subjected to mapping. **c** Alternatively, after treatment with PRP for 60 min, the plates with adherent platelets were fixed, dehydrated, and examined using EDS. Blue, red, and green dots represent Ti, Ca, and P, respectively. Adherent platelets are faint yellow

Figure 3 shows the elemental mapping of the surfaces of plain and CaP-blasted cp-Ti plates. Because the plain cp-Ti plate was not chemically or mechanically mirror-finished, relatively large (micron order) yet gentle roughness was observed on the surface. In addition, Ti (indicated by blue dots) was detected as a major element on the surface. After CaP-blasting, sub-micron levels of roughness were formed on the surface, and some areas were coated with Ca (red) and P (green). Under these blasting conditions, the area ratio of Ti to CaP was approximately 50:50. In addition, after incubation with PRP for 60 min, the platelets adhered to the surface mainly by means of their pseudopodia, regardless of the distribution of surface elements or topography (convex or concave).

**Fig. 4:**
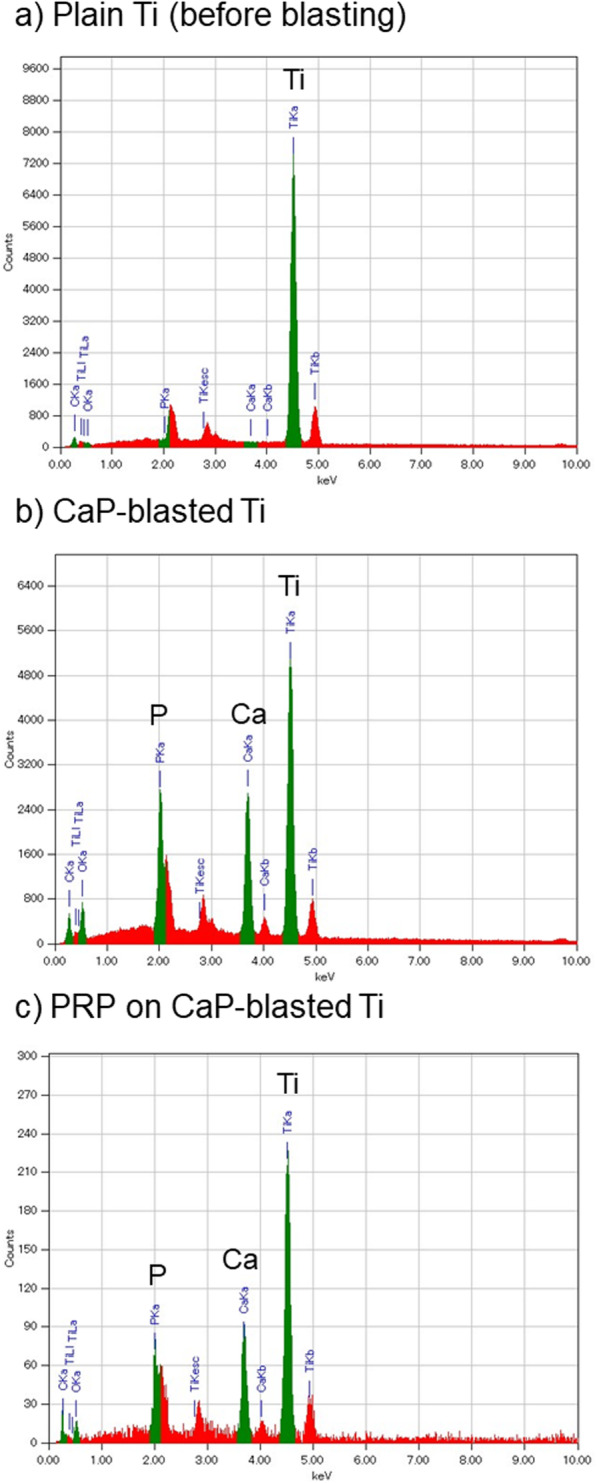
Elemental analysis of plain and CaP-blasted cp-Ti plate surfaces. **a** Plain cp-Ti plate before blasting, **b** CaP-blasted cp-Ti plate, and **c** PRP-treated, CaP-blasted cp-Ti plate

Figure 4 shows the elemental analysis of the plain and CaP-blasted cp-Ti plate surfaces. In the plain cp-Ti surface used for blasting, Ti was the only major element among the elements tested (Ti: approximately 8000, Ca: < 100, P: < 100 counts) (Fig. 2a). CaP-blasting increased Ca and P contents (Ca: approx. 2700, P: 2800 counts), but reduced apparent Ti contents (approx. 5000 counts), and the ratios of Ca and P to Ti were roughly 0.54 and 0.56, respectively (Fig. 2b). Due to the adhered platelets, adsorbed plasma proteins, and other components on the CaP-blasted cp-Ti surface, incubation with PRP substantially reduced the apparent counts of these elements (Ti: approx. 230, Ca: 80, P: 70 counts) (Fig. 2c). Nevertheless, compared with the non-blasted cp-Ti surface (Fig. 2a), the ratios of the elements (Ca/P: 0.34, P/Ti: 0.30) seemed similar to those on the same plate without incubation with PRP. It is noted that PRP with relatively low platelet counts were used to avoid full coverage of the surface by platelets.

**Fig. 5:**
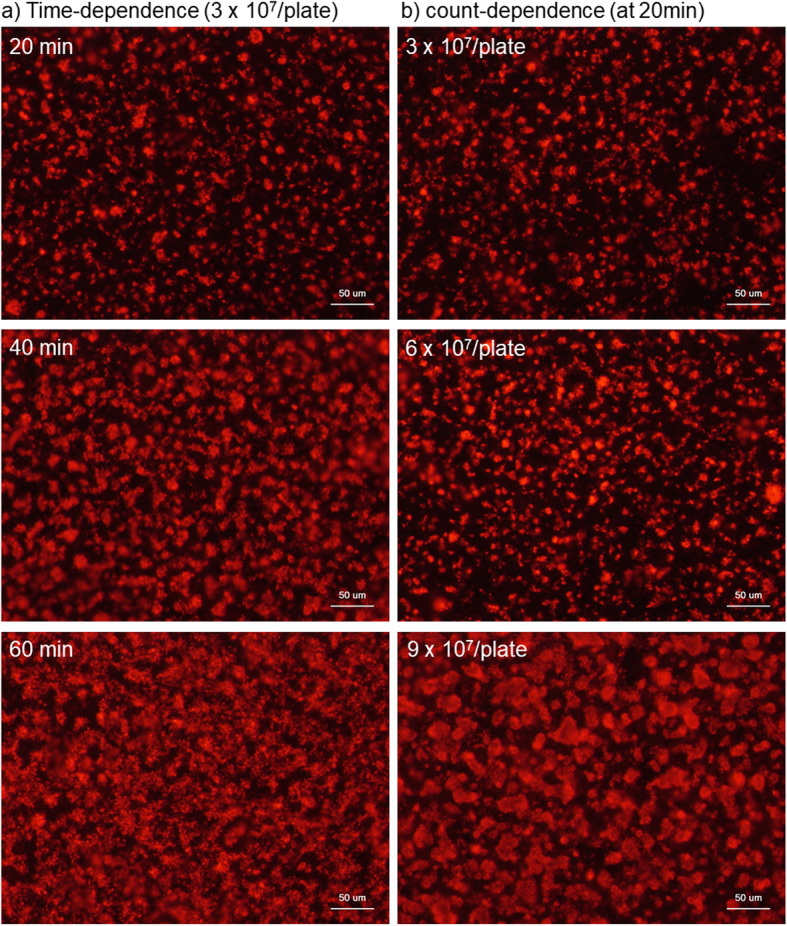
Time- and count-dependent platelet adhesion on CaP-blasted cp-Ti plates. **a** Platelets in the form of PRP was inoculated at the fixed count of 3 × 10^7^/plate and incubated at ambient temperature for 20, 40, and 60 min. **b** Platelets in the form of PRP was inoculated at counts of 3 ×, 6 ×, and 9 × 10^7^/plate and incubated for the fixed period of time, 20 min. Cytoskeletal actin filaments were stained with phalloidin

Figure 5 shows the time- and count-dependent platelet adhesion on CaP-blasted cp-Ti plates. When platelets were inoculated on the plates at a count of 3 × 10^7^/plate, platelet adhesion increased in a time-dependent manner. After 60 min of incubation, the plate was almost fully covered by platelets. When compared at 20 min of incubation, platelet adhesion increased in a platelet-count-dependent manner. At a count of 9 × 10^7^/plate, the plate was almost fully covered by platelets.

**Fig. 6:**
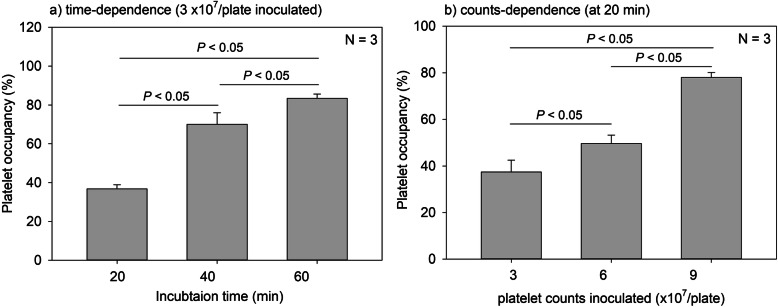
Time- and count-dependent platelet occupancy on CaP-blasted cp-Ti plates. The images obtained by actin staining were subjected to image analysis and platelet occupancy against the fixed region of interest was calculated. *N* = 3

Figure 6 shows the time- and count-dependent platelet occupancy on CaP-blasted cp-Ti plates. The number of platelets adhered on CaP-blasted cp-Ti plates increased with time (20 min: 36.8 ± 2.1%, 40 min: 70.0 ± 6.0%, 60 min: 83.4 ± 2.2%) and inoculum size (3 × 10^7^/plate: 37.5 ± 5.1%, 6 × 10^7^/plate: 49.7 ± 3.6%, 9 × 10^7^/plate: 78.1 ± 2.1%). The images that the plates were almost fully covered by platelets were evaluated by the image analysis to be approximately 80% occupancy.

**Fig. 7:**
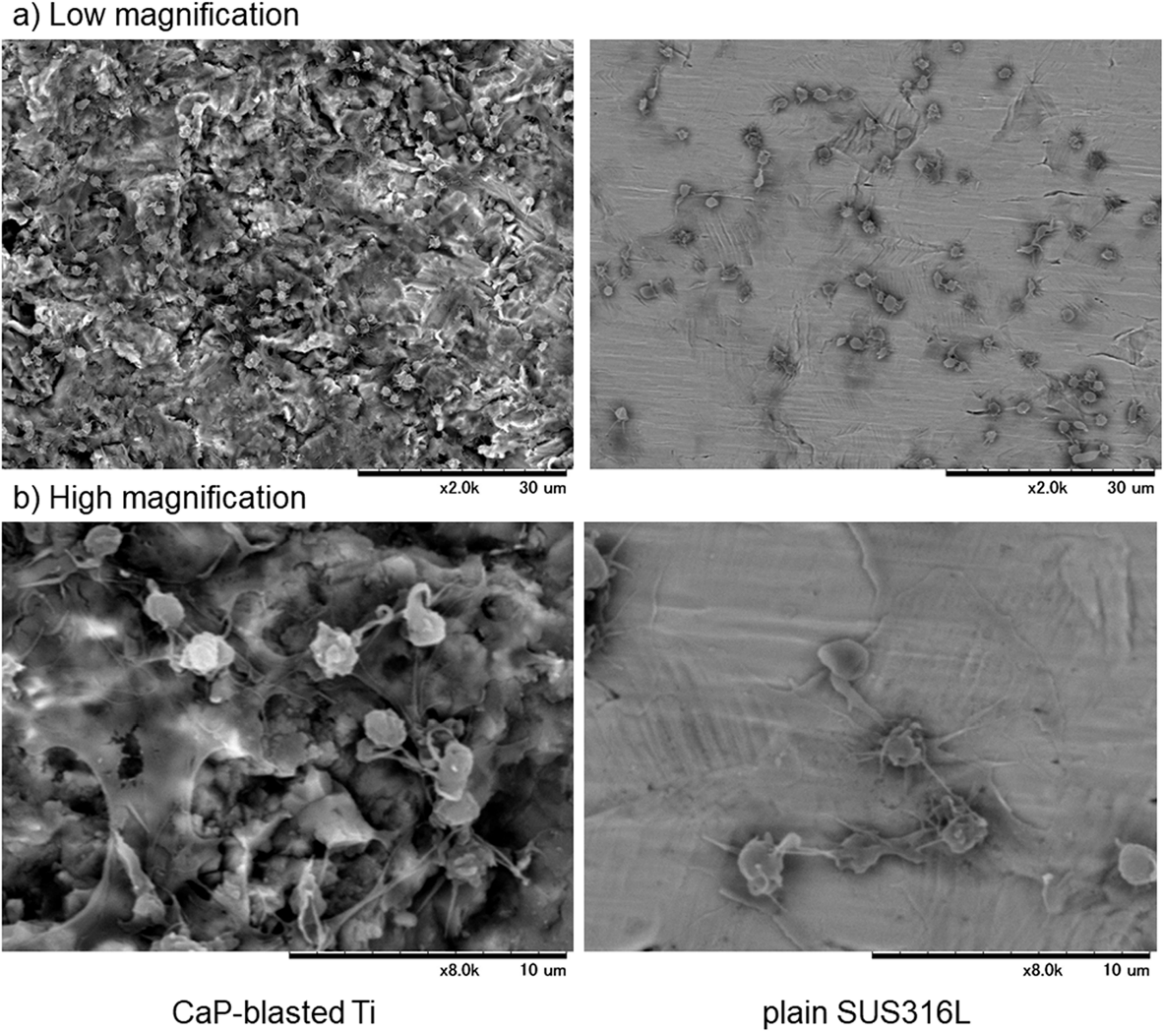
SEM observations of platelets adherent on CaP-blasted cp-Ti and plain SUS316L plates. PRP was placed on the plates, incubated for 60 min, washed, fixed, and dehydrated for SEM examination. **a** Low magnification and **b** high magnification

Hereafter, the data are expressed by comparison with plain SUS316L plates, which are widely used in the production of medical devices, including injection needles. For reference, platelet adhesion and reaction on plain cp-Ti plates have been vigorously reported in previous studies [2, 16]. Platelet density and morphology were examined using SEM at low and high magnification. Figure 7 shows SEM observations of platelets adhered on CaP-blasted cp-Ti and plain SUS316L plates after 60-min incubation. In CaP-blasted cp-Ti plates, when PRP with relatively high platelet counts was used (vs. Fig. 3), platelets adhered to both the convex and concave surfaces of the micro-topography at higher densities. Some platelets formed pseudopodia; however, the majority of adherent platelets were spherical. In contrast, on plain SUS316L plates, platelet density was seemingly much lower than that on CaP-blasted cp-Ti plates, and many platelets adhered onto the surface by spreading their pseudopodia.

**Fig. 8:**
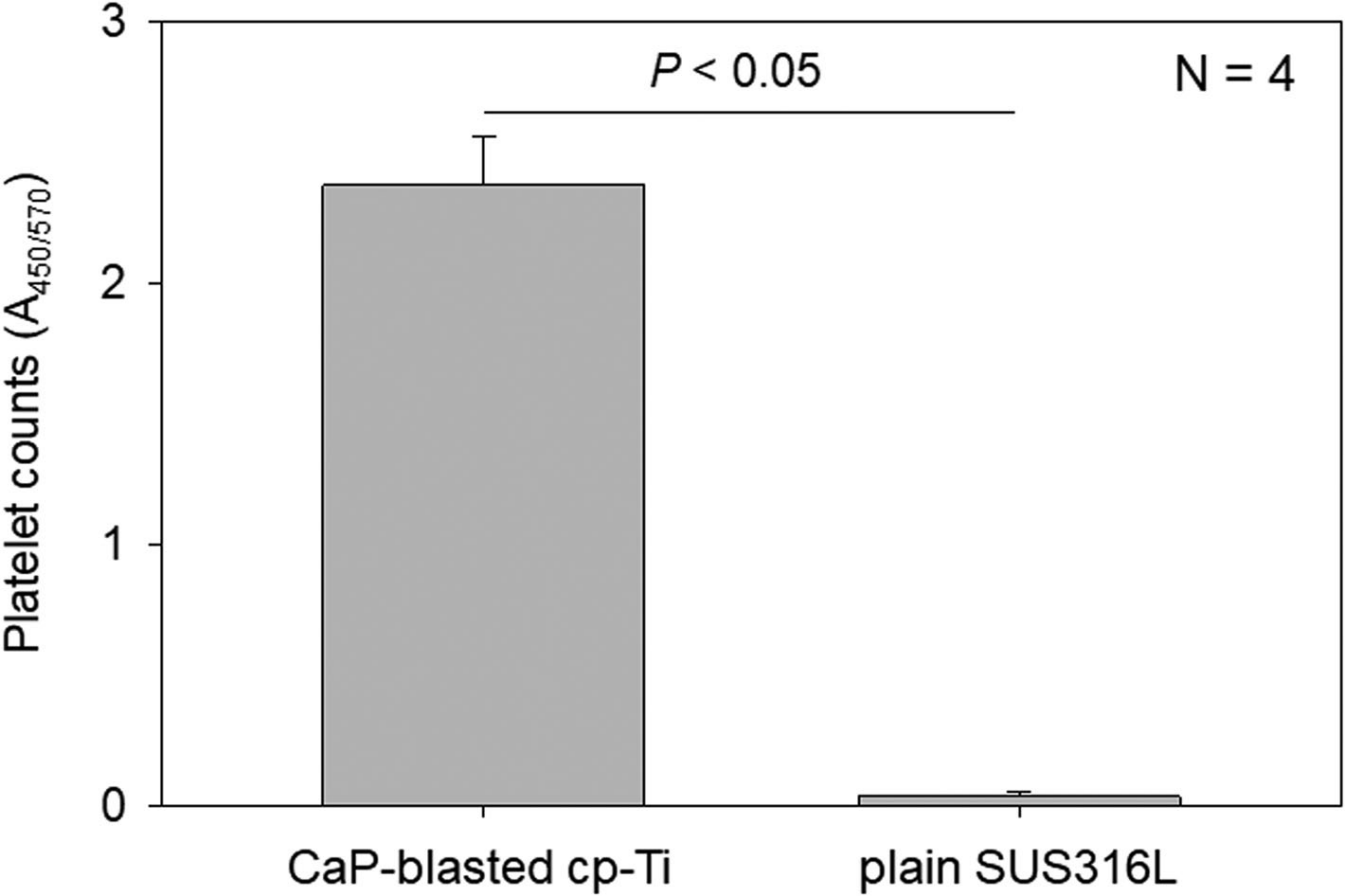
Number of platelets adherent on CaP-blasted cp-Ti and plain SUS316L plates. After incubation for 60 min, the plates were rinsed and further incubated with CCK-8 for 90 min. The amount of converted formazan was quantified using a microplate reader at 450 nm. *N* = 4. *P* < 0.05 compared to SUS316

To confirm the difference in platelet density, platelet counts were quantified using a tetrazolium salt. Figure 8 shows the number of platelets adherent on CaP-blasted cp-Ti and plain SUS316L plates. Because we prepared pure PRP, significant numbers of white and red blood cells were excluded from the resulting pure PRP preparations. Even though some white and/or red blood cells were included, such contamination is not believed to significantly disturb platelet counts. The spectrophotometric assay demonstrated that considerably greater levels of platelets were entrapped and adherent on the CaP-blasted cp-Ti plates (*A*_450/570_: 2.373 ± 0.187), as compared to plain SUS316L plates (*A*_450/570_: 0.037 ± 0.019) (*P* < 0.05).

**Fig. 9:**
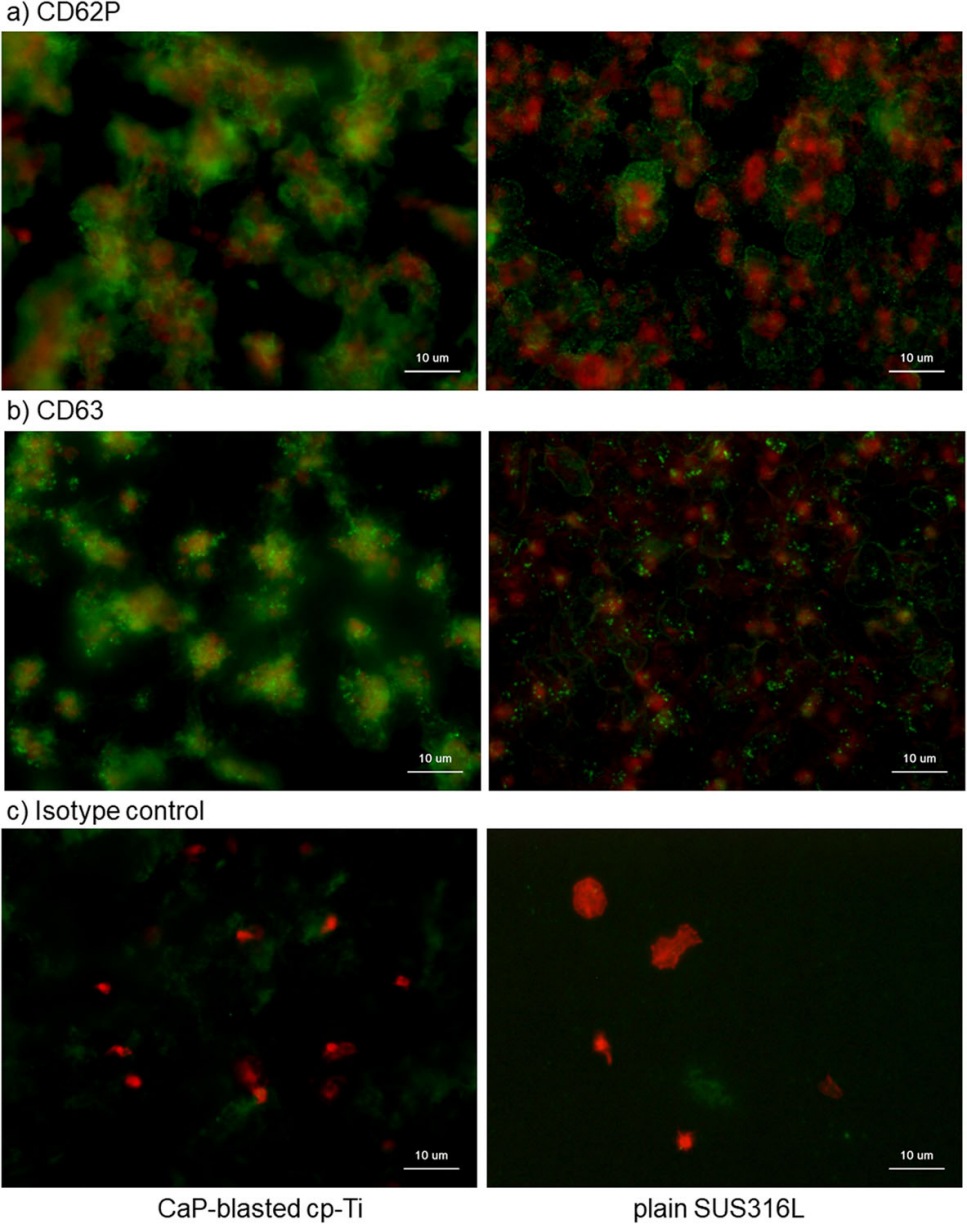
Immunocytochemical detection of activated platelets expressing **a** CD62P or **b** CD63. CaP-blasted cp-Ti and plain SUS316L plates were incubated with PRP for 60 min, washed, fixed, and subjected to immunocytochemical visualization. Green represents proteins reactive for **a** CD62P or **b** CD63, while red represents polymerized actin. **c** To show non-specific detection in extra-platelet spaces, PRP containing relatively low platelet counts was added on the plates and examined

Figure 9 shows immunocytochemical detection of activated platelets that express CD62P and CD63. In the negative control (using non-immunized mouse IgG), faint non-specific detection was observed: however, this level was substantially lower than that required for specific detection. The expression levels of both CD62P and CD63 (green) were substantially upregulated in individual platelets (which were marked by cytoskeletal actin fibers shown by red) on CaP-blasted cp-Ti plates, as compared to plain SUS316L plates. This implies that platelets adherent on CaP-blast cp-Ti plates are activated at higher levels.

**Fig. 10:**
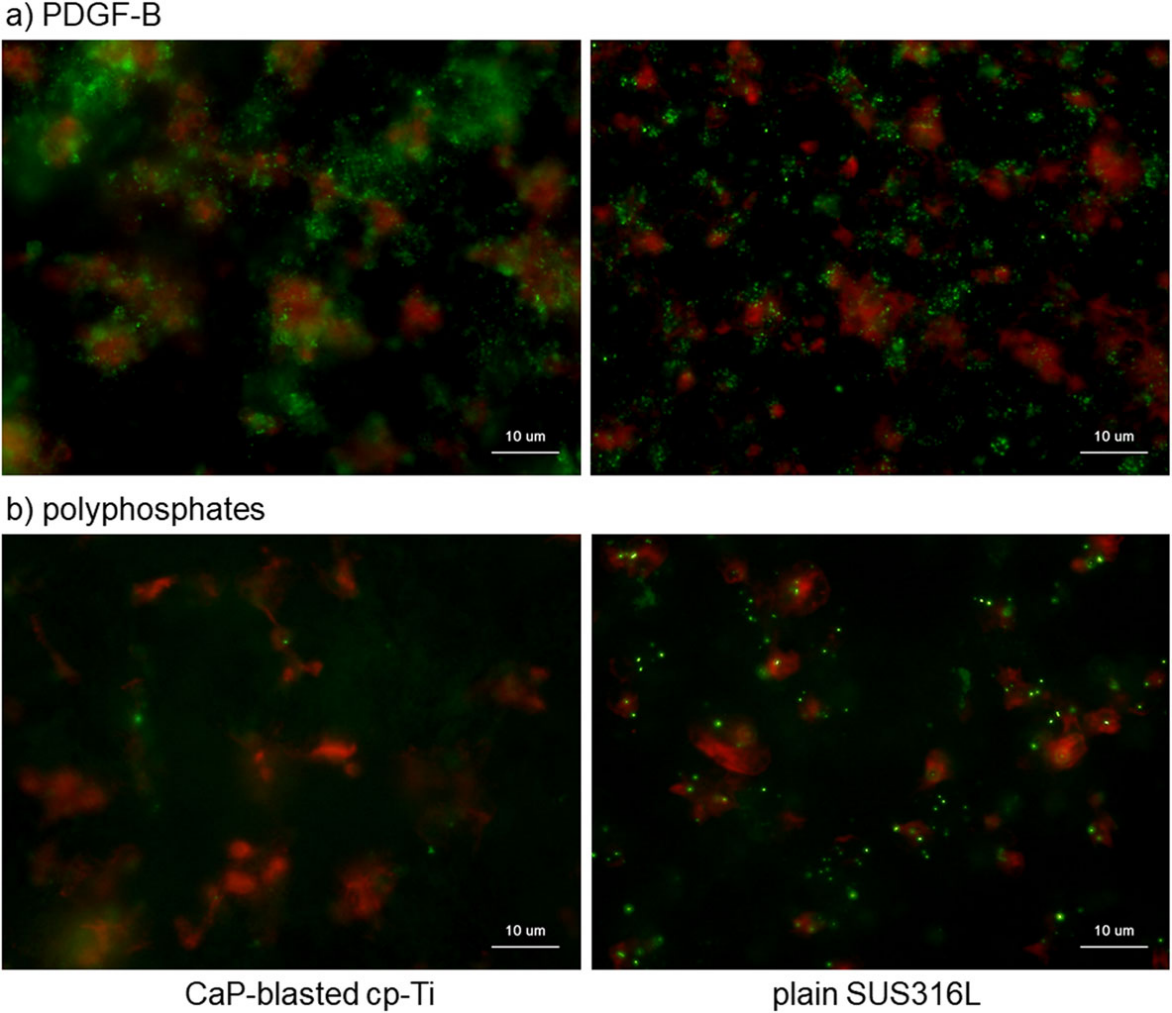
**a** Immunocytochemical visualization of PDGF-B and **b** chemical detection of polyphosphates. CaP-blasted cp-Ti and plain SUS316 plates were incubated with PRP for 60 min, washed, fixed, and subjected to immunocytochemical or chemical detection. Green represents proteins reactive for PDGF-B (**a**) or polyphosphates (**b**), while red represents polymerized actin

Since activated platelets release biomolecules, such as growth factors, the representative growth factors PDGF-B and polyphosphates were subsequently visualized. Figure 10 shows the immunocytochemical visualization of PDGF-B and the chemical detection of polyphosphates. On CaP-blasted cp-Ti plates, PDGF-B was detected mainly in extra-platelet spaces, whereas this growth factor was colocalized with cytoplasmic actin fibers or found in extra-platelet spaces on plain SUS316L plates. In contrast, DAPI-reactive particles (green), that is, polyphosphates, were found in platelets on plain SUS316L plates, while DAPI-reactive polyphosphates were widely diffused in the form of smears within extra-platelet spaces (faintly stained by green).

## Discussion

This study demonstrated that CaP-blasted cp-Ti plates are more efficient in the entrapment and activation of platelets compared to plain cp-Ti plates examined in previous studies [2, 16]. Thus, CaP-blasted Ti implants could be functionalized with platelets and biomolecules provided by PRP to contribute to more significant regeneration of surrounding tissues and consequent osseointegration. Although further studies are required, such as to validate these findings in animal models, the present study provides evidence that supports the PRP immersion of implants prior to application in clinical settings and suggests that as necessity, the period of immersion time can be modulated by platelet counts in individual PRP preparations (for a more detailed discussion, see the subsection “Clinical Relevance”).

### Platelet adhesion and activation

The differences in platelet adhesion between CaP-blasted cp-Ti and plain SUS316L plates were speculated to be due to variations in surface wettability, energy, roughness, and/or chemical composition [15, 23]. As reported previously [5], CaP-blasting enlarges the free surface area, increases the surface roughness, and enables the attachment of CaP (which serves as an abrasive). Among these factors, chemical composition is the well-known to influence protein adsorption. In general, CaP surfaces adsorb a greater amount of protein than Ti surfaces [24]. Therefore, it could be hypothesized that platelets adhere more efficiently to the surface of CaP-blasted cp-Ti plates. As expected, we found that a greater number of platelets were entrapped on the surface of CaP-blasted cp-Ti plates than on the surface of plain cp-Ti plates (as well as plain SUS316L plates), even when treated in the form of PRP.

However, since we preliminarily observed that platelets were entrapped at much lower levels on decalcified blasted cp-Ti plates, i.e., CaP-free, blasted cp-Ti plates, the efficient entrapment may not be solely due to the specific surface micro-topography or enlarged surface area. This finding is consistent with that of a previous study [25]. However, it cannot be ruled out that decalcification with EDTA may also influence surface chemistry and/or wettability and that micro-topography may function synergistically with CaP-coating, as described below.

We also examined other factors in this study, such as wettability, and found no significant differences between the CaP-blasted cp-Ti and plain SUS316L plates. This observation could be explained by the data described below. CaP and HA, alone or in combination, display high wettability (contact angle: 40–60°) [26], and the contact angle of cp-Ti is 55-60° [27]. In addition, despite the use of a Ti alloy (Ti-6Al-4V), surface roughness has a weak impact on wettability [28].

Recently, numerous studies have vigorously investigated the adsorption of proteins on CaP surfaces [29–32]. In a previous study [2], we demonstrated the involvement of adhesion molecules adsorbed on plain cp-Ti plates in platelet adhesion. Thus, adhesion molecules, such as fibronectin and von Willebrand factor, may potentially get adsorbed at higher levels on CaP-blasted cp-Ti surfaces than on plain surfaces, which allows CaP-blasted cp-Ti surfaces to gather platelets more efficiently. However, it still remains to be clarified the reason why other plasma proteins, such as albumin, do not disturb the platelet adhesion, as observed on the plain surface [2]. Thus, further investigation is needed to clarify the mechanisms responsible for enhanced cell adhesion and protein adsorption on the CaP-blasted cp-Ti surfaces, as compared to plain surfaces.

Platelets are naïve cells that can be easily activated by various mechanical and chemical stimuli, such as centrifugation, and contact with low-biocompatible materials, fibrin, and adenosine diphosphate. In previous studies [2, 16], we demonstrated that platelets served in the form of PBS suspension are significantly activated to upregulate CD62P and CD63 expression and growth factor release on the CaP-blasted cp-Ti surface, compared to on the plain cp-Ti surface. Thus, although the mechanisms for this effect are not fully understood, it is evident that adhesion by itself activates platelets and potentially vice versa.

Differences in surface topography render it difficult to compare the levels of platelet activation between plain and CaP-blasted cp-Ti plates simply by means of image analysis. However, platelets seemed to be more activated on the CaP-blasted cp-Ti surface, rather than on the plain surface. This may be due to enhanced platelet adhesion on the CaP-blasted cp-Ti surface.

### Biomolecule release and entrapment in extra-platelet spaces

It is well known that activated platelets degranulate α- and dense granules [33]. In this study, PDGF-B (and PDGF-B-like proteins), which are stored in α-granules [34], were localized mainly in platelets on the plain SUS316L surface. On the other hand, they were widely distributed in the extra-platelet spaces on the CaP-blasted cp-Ti surface.

Polyphosphates are stored in dense granules and are released upon activation [35]. Thus, it is anticipated that similar to PDGF-B, polyphosphates would also be distributed in extra-platelet spaces upon activation. In this study, polyphosphates were visualized as particles in platelets on the plain SUS316L surface, and their distribution was similar to that of PDGF-B. In contrast, on the CaP-blasted cp-Ti surface, polyphosphates diffused, and thus faintly observed. Due to their nature, visualization and quantification of polyphosphates is not yet established, and is consequently limited, especially in terms of sensitivity [36]. However, recent advances have enabled the visualization of polyphosphates using DAPI, specifically by switching excitation and/or emission wavelengths [37]. We are still undergoing the process of further optimizing the experimental protocol; however, for the first time, we have successfully demonstrated that polyphosphates are released from activated platelets and diffuse, becoming diluted as they enter extra-platelet spaces.

It has generally been accepted that polyphosphates are involved in various platelet functions, such as coagulation [38]. In addition, their involvement in biomineralization has increasingly been focused on in the past decade [39–43]. Therefore, to comprehensively understand the potential of PRP therapy, it is necessary to investigate the roles of polyphosphates more vigorously.

### SUS316L stainless steel plates

SUS316L is a subtype of stainless steel that is widely used in medical devices, including injection needles. Laboratory technicians routinely use plastic pipettes for PRP preparation, while many clinicians and operators working in private practices prefer using syringes with injection needles, especially non-bevel needles. In the process of blood collection, the blood passes though these needles. It is believed that blood coagulation starts upon contact of platelets and coagulation factors with the inner walls of the needles when blood passes through them; thus, large bore needles are advised to be used to avoid unintentional activation of platelets [44, 45]. However, our present data suggest that the plain SUS316L surface may not significantly influence platelet activation, as long as relatively large bore needles are used. In addition, there may not be a significant loss in platelet levels in the presence of anticoagulants.

### Clinical relevance

According to Davis [46, 47] and Junker et al. [10], among the series of events occurring between the host and the implant surface immediately after implant placement, the first and the most important healing phase is osteoconduction. This process promotes the directed growth of osteogenic cells through platelet activation, subsequent osteogenic cell migration, and fibrin clot stabilization on rough surface. Thus, we expected that this phase was facilitated or augmented by coating with the implant with PRP. Nikolidakis et al. [48, 49] demonstrated in an animal-based study that PRP in liquid form (without activation) increases bone apposition on CaP-coated rough surface Ti implants. In this context, we suggest that the PRP immersion of CaP-blasted Ti implants prior to clinical application may be beneficial for initial implant stability.

To date, however, the subsequent preclinical and clinical studies have provided contradicting findings [50–54] and the effects of PRP remain controversial. Since implant osseointegration is influenced by several factors such as implant design, implant surface, surgical technique, bone type, and loading conditions in addition to the varied quality of individual PRP preparations [55], we should not conclude that PRP treatment does not benefit osseointegration or facilitate initial implant stability. In view of the current scenario, the European Association for Osseointegration, on its website, replies to clinicians’ questions regarding the effects of PRP on implant success rates [56]. Neither PRP nor plasma-rich growth factors (PRGF) have been shown to improve implant stability or reduce marginal bone loss following implant placement. Although a randomized clinical trial was carried out, currently there is insufficient evidence to support a clinical recommendation on this basis [55].

This in vitro study focused on the effects of PRP immersion on implant surfaces using a small sample size (*N* = 6) by limiting sample donors, as followed in our previous studies (*N* = 2–15) in the past 2 years [2, 16, 20, 21, 57–64]. Thus, to evaluate the population type (Gaussian or not), individual differences, and spread of distribution, or to more strictly validate and generalize these results, further comparative studies with larger sample sizes are warranted that should include female donors.

## Conclusions

Owing to their unique surface properties, CaP-blasted cp-Ti plates are capable of efficiently inducing platelet adhesion and activation to release biomolecules involved in regeneration of the surrounding tissue. Therefore, immersion of this type of dental implant in liquid PRP could contribute to the augmentation of their initial stability.

## Data Availability

The data are available from the corresponding author on reasonable request.

## Abbreviations

PRP: Platelet-rich plasma
ACD-A: A formulation of acid-citrate-dextrose
cp-Ti: Commercially pure titanium
PBS: Phosphate-buffered saline
HA: Hydroxyapatite
β-TCP: β-Tricalcium phosphate
CaP: Calcium phosphate
T-PBS: 0.1% Tween-20-containing PBS
DAPI: 4′,6-Diamidino-2-phenylindole
